# Supplementary material for: A mechanistic theory of planning in prefrontal cortex
Source: eLife. 2026 Oct 21;15:RP109757. doi: 10.7554/eLife.109757 (PMC13686619; doi:10.7554/eLife.109757)
Supplement: Supplementary file 2 [file elife-109757-fig1.docx]

00:00:00
In this video, I explain figure one of the paper, where we discuss relevant background work that inspired our research.
The first thing we consider in panel A is sequence representation to prefrontal cortex.
Recent research showed that when an animal has to keep a sequence of actions in working memory, different neural subspaces represent each element of the sequence.
This means that if an animal has to represent the sequence to go from here through these intermediate locations to the goal, different populations of prefrontal neurons will represent the first location, the second location, the third, and the fourth location of the sequence.
So here, four neurons are active all at the same time, and together they concurrently represent the entire sequence of actions to the goal.

00:00:36
We'll combine these ideas with the idea of neural networks performing inference.
We can illustrate this with an example from visual cortex.
If you show an animal this stimulus here, it immediately infers the presence of a square that doesn't actually exist.

And individual neurons in the primary visual cortex will fire at a high rate if you align their receptive field to the inferred line, which is not true for this control stimulus.
So here, the brain uses partial noisy information to infer the presence of an entire square that doesn't really exist in the world.
Similarly, in head direction circuits, neural networks or head direction circuits receive noisy information about self-motion and vision, and this is combined with structural knowledge embedded in synaptic connectivity to infer the current orientation of the animal.

00:01:18
We'll show that if we combine these ideas of inference in neural networks with these recently discovered prefrontal sequence representations, it give--gives rise to a powerful planning algorithm that allows networks to solve complex planning problems.
We'll also see that this algorithm, which we call the space-time attractor, is particularly well-suited to the kinds of problems that prefrontal cortex is important for.
This is relevant because there are different types of tasks that depend more or less on the PFC.
In particular, what we call static tasks are less dependent on the prefrontal cortex.
This is where you always take the same action in response to the same stimulus.

00:01:51
You always pull the lever whenever you see the slot machine.
Conversely, PFC is more important for what we call dynamic tasks.
This is when you have to take different actions depending on the context.
For example, if you have a cinema ticket for nine PM, but you want to go for dinner first, you need to be at the cinema at nine, not at ten, you'll have missed the film, and not at eight, because you'll be there way too early.

And this impacts when you should go for dinner at the restaurant as well.
So in this case, your action, whether to go to the cinema or the restaurant, depends on the context, in this case, the time of the day.
We'll see that this space-time attractor is particularly well-suited to these dynamic tasks, where simpler algorithms can solve these static tasks.
