# Supplementary material for: A mechanistic theory of planning in prefrontal cortex
Source: eLife. 2026 Oct 21;15:RP109757. doi: 10.7554/eLife.109757 (PMC13686619; doi:10.7554/eLife.109757)
Supplement: Supplementary file 3 [file elife-109757-fig2.docx]

00:00:01
In this video, I explain figure two of the paper, where we explain how the space-time attractor works by comparing it to other attractor networks known in the brain.
In panel A, we first show a ring attractor.
This is a network where different neurons have different preferred orientations, and the connectivity embeds the structure of the world such that nearby orientations excite each other and distant orientations inhibi-inhibit each other.
This connectivity constrains the possible stable representations or fixed points of the network to only represent single orientations.

00:00:33
So one fixed point might represent facing west, while another one would represent facing east.
Grid cells can be implemented through a similar principle.
In this case, neurons excite each other if they represent nearby locations in Euclidean space, and they inhibit each other at intermediate distances.

This constrains the fixed points or the stable representations to be these hexagonal patterns of activity known from grid cells.
The space-time attractor is a three-dimensional network.
In this case, neurons care about both the location in space and the time in the future.

00:01:06
The relevant structure that needs to be embedded in the connectivity is which locations can form part of the same trajectory.
This means that neurons excite each other if they represent a plan to be at adjacent locations in space at adjacent moments in time.
In this case, the connectivity constrains the possible fixed points to be continuous trajectories in space and time.
And here in the space-time attractor, the fixed points are input-dependent.
So given this connectivity and an input that says where you are and where you're trying to go, the fixed points will represent trajectories from that specific location to that specific goal location.

00:01:42
If you change the input with the exact same connectivity, the fixed point would now represent the trajectory from the new location to the new goal.
This means you can start from some initial noisy activity pattern, and you can just provide inputs that say where you are and what you're trying to achieve.
And then when that's combined with this specific structure connectivity, the representation will slowly converge to a plan that says where you should be at which point in time.
This allows the animal to read out a policy, what it should be doing next, which is simply the state represented in the immediate future population of neurons.
When it moves to this new location, that's gonna update the input to the network, which is gonna change the fixed point and shift the activity to tell the animal what to do next.
