# Supplementary material for: A mechanistic theory of planning in prefrontal cortex
Source: eLife. 2026 Oct 21;15:RP109757. doi: 10.7554/eLife.109757 (PMC13686619; doi:10.7554/eLife.109757)
Supplement: Supplementary file 4 [file elife-109757-fig3.docx]

00:00:01
In this video, I explain figure three of the paper, where we show the dynamics and fixed points of the space-time attractor.
We start by focusing on this fully connected environment, where the animal starts in the lower-left corner, and there's a goal in the upper-right corner.
What we show is the representation that tells the agent where it expects to be right now and at different times in the future.
And on the right, we show a max projection across these populations, which can be seen as a summary that says where it will be at any time in the future.
We start from this empty uniform representation and see that information gradually propagates from the current location and the goal to intermediate representations or intermediate times in the plan, until the network eventually converges to a stable representation of where it should be at different times in the future in order to reach the goal as soon as possible.

00:00:47
We then consider an environment where there are two equivalent but disjoint paths to the goal.
We again start from the uniform representation and now see that at intermediate times, these two possible paths are competing in the network.
But due to the competitive winner-take-all dynamics of this attractor network, eventually, just one of them wins out, and it tells the animal what it should do next.
However, both of these two representations, both of these two paths are fixed points of the network dynamics, so if we re-run the noisy dynamics in a second trial, we might converge to the other representation instead.

00:01:19
And both of these representations are reached at approximately equal frequencies.
We then consider what happens if we take away the input from the network, if we no longer tells it where it's trying to get to, right?
So we keep the location input, but we remove the goal input.

In this case, the network relaxes back to this diffusive representation that just says the animal will probably be at nearby locations to where it started out.
This illustrates the input dependence of the fixed points in the s-in the space-time attractor.
The connectivity tells the network what possible representations are, that it's constrained to represent continuous trajectories, but the inputs tell it which trajectories are relevant in a particular context.

00:02:01
Finally, we consider this environment where there are two paths of different length to the goal.
In this case, the network almost always converges to the shortest path if we initialize it from the uniform representation.
So in this case, the shortest path has the largest basin of attraction in the attractor network.
However, the other path is also a fixed point of the network dynamics, so if we were to initialize the network so it's closer in trajectory space to this long path, it might fall into that particular attractor state and eventually represent the longer path to the goal and tell the animal it should go this way instead of this other way.
