# Supplementary material for: A mechanistic theory of planning in prefrontal cortex
Source: eLife. 2026 Oct 21;15:RP109757. doi: 10.7554/eLife.109757 (PMC13686619; doi:10.7554/eLife.109757)
Supplement: Supplementary file 5 [file elife-109757-fig4.docx]

00:00:01
In this video, I explain figure four, where we compare the space-time attractor to two baseline algorithms commonly studied in the neuroscience literature.
We do so across tasks that vary from more static to more dynamic.
An example of a static task is where there's a fixed goal that remains in the same location across trials, and the agent has to navigate to this goal location from different locations in the environment.
We can make this task more dynamic by allowing the goal to move from one trial to the next, so the agent has to navigate to the trial-specific goal location.

00:00:33
We can then let the goal move not just across trials, but also within a trial.
So it starts out here, but then it follows this gray trajectory as the animal moves around the environment.
Now, the optimal policy is not to move to where the goal is now, but where it will be in the future.

We can generalize this idea of time-dependent reward functions in what we call the reward landscape task.
In this case, we sample a different reward for every location in space, and we resample that reward function again at every moment in time.
The goal of the agent is to maximize cumulative reward, which can require the agent to move to a low reward location in the immediate future because it gives access to a high reward location in the more distant future.

00:01:14
We compare the space-time attractor to two baseline algorithms, a temporal difference learner, which slowly generates or computes a value function across many trials, and a successor representation agent, which estimates its expected future occupancy and combines that with the trial-specific reward function to compute a trial-specific value function.
In the fixed static goal task, both of these agents can compute a correct value function, which tells the agent where to go next.
The space-time attractor can also solve this task, but it does so in a very different way.
It receives input that says where the agent is and where the goal is, and the recurrent dynamics infer an explicit representation of a future trajectory to the goal.

00:01:53
We can quantify the performance by computing the probability that each of these agents takes an optimal first action within each trial, and we see that all of, all three algorithms perform essentially optimally compared to a random baseline and a greedy baseline.
This changes in the changing static goal task, where the successor representation agent continues to perform well, but the temporal difference agent struggles because it has to combine experience across different trials, but the goal location moves between trials.
The space-time attractor also continues to solve this task, again because it has access to trial-specific reward information.

00:02:30
And when we quantify it, we see that the successor representation agent and the space-time attractor continue to perform optimally, while the temporal difference agent does not.
We then move to the moving goal task, and in this case, the successor representation agent also struggles.
The reason for this is that it computes an expected future occupancy averaged across time, so the SR agent doesn't have access to detailed information about when it will be where, and it can only compute an average value function across the entire trial.
This is different than the space-time attractor because it has an explicit representation of what happens at which moment in time, and that allows it to infer a trajectory that intercepts the goal at the correct location in space and time.

00:03:12
We can again quantify it and see that the STA is the only algorithm that continues to perform well in this moving goal task.
This difference gets even larger in the reward landscape task, where there are weaker correlations between the reward function in space and time.
So what these results suggest is that the space-time attractor is particularly suited to these types of dynamic tasks that we also know depend critically on the prefrontal cortex.
