# Supplementary material for: A mechanistic theory of planning in prefrontal cortex
Source: eLife. 2026 Oct 21;15:RP109757. doi: 10.7554/eLife.109757 (PMC13686619; doi:10.7554/eLife.109757)
Supplement: Supplementary file 6 [file elife-109757-fig5.docx]

00:00:01
In this video, I explain figure five, where we show that recurrent neural networks learn explicit representations of the future when they're trained to solve challenging planning tasks.
In panel A, we first illustrate what these representations look like.
When an agent is in some location and trying to move to a goal, we expect that different populations of neurons or different patterns of neural activity should represent the entire future of the agent.
We also know how these representations should update.
When the agent moves to the next location, neural activity should update to represent the future to come.

00:00:35
Importantly, these representations should generalize across different trajectories.
So if there's a different trial where the animal or the agent comes from a different location, but it's gonna visit the same location in two actions time, then that particular subspace of neural activity should generalize across the first action of trial two and the second action of trial one.
We're going to compare three different agents.
The first is the handcrafted space-time attractor from before.
The second is a recurrent neural network that's trained to optimally solve the reward landscape task.

00:01:09
And the third is what we call a value-based agent, which uses dynamic programming to compute a value function in space and time, and then moves to the adjacent location with the highest value.
We start by focusing on what we call the planning period, which is the neural representation before the agent takes the first action.
We then train decoders to predict where the agent will be at different times in the future.
These decoders are trained in cross-validation, so we train them when the agent is at every location apart from one, and then we then test on the held-out location.

00:01:41
We see that both the handcrafted STA and the task-optimized RNN learn representations of where it will be at each time in the future, which is not true for the value-based agent.
And these two agents also learn to represent when it will be at a specific location.
We then focus on the execution period, where we find that both the STA and the task-optimized RNN have much stronger representations of where they will be in the future than where they were in the past, which is reminiscent of recent experimental data.
Finally, we ask how these representations update.

00:02:14
To do so, we train a single de-decoder to predict where the agent will be at time t equals three from the neural activity at time t equals one.
We then ask how this decoder generalizes.
When we apply the same decoder to neural activity at t equals zero, it suddenly predicts location at t equals two.
And when we apply it to neural activity at time t equals two, it predicts neural activity at t equals four.
What this shows is that there's an invariant subspace of neural activity that predicts where the agent will be in delta actions time, which are consistent between the handcrafted space-time attractor and the task-optimized RNN, and which is different from this value-based agent that instead has an invariant representation of where the agent will be at a fixed moment in time.

00:03:01
Finally, we compare recurrent neural networks trained on different tasks.
We train the RNNs either on the static-goal task, the moving-goal task, or the reward landscape task, and we then ask how well they solve each task.
We find that they can all solve the static-goal task, which is perhaps not surprising because it's a special case of the moving-goal and the reward landscape task.
When we move to the moving-goal task, the RNN trained on the static goal suffers, and when we move to the more difficult reward landscape task, only the agent trained on the reward landscape task is able to solve it.

00:03:33
This is again not too surprising, but it does illustrate that networks only learn this complicated solution of planning as inference through explicit future representations when it's actually necessary to solve the task, and these simpler tasks can be solved using simpler algorithms.
Additionally, there's a pressure to do so because if we compare the cost of implementing these different algorithms, we find that both, both the magnitude of the weights of the neural network and the average firing rates are much higher for the RNN trained on the reward landscape task, even when applied to the simpler tasks.
So what this tells us is that when recurrent neural networks are forced to solve complicated planning tasks with reward functions that change rapidly in space and time, then there's a pressure for them to learn this complicated solution, which is fairly expensive but allows it to generalize across many different tasks.
Whereas if a network only ever has to solve the simpler tasks, then it can learn much simpler solutions as well.
