# Supplementary material for: A mechanistic theory of planning in prefrontal cortex
Source: eLife. 2026 Oct 21;15:RP109757. doi: 10.7554/eLife.109757 (PMC13686619; doi:10.7554/eLife.109757)
Supplement: Supplementary file 7 [file elife-109757-fig6.docx]

00:00:01
In this video, I explain Figure six, where we show that recurrent neural networks learn to embed the structure of the world in their connectivity.
We do so by taking the neural activity of a trained RNN, and we project it into what we call a space-time coordinate system.
We do so by finding orthogonal directions in neural state space that predict being at different locations in space at different moments in time.
This is useful because we can project the parameters of the neural network into this coordinate system, and we can then interpret the resulting projected parameters as interactions between different points in space-time.

00:00:36
And that allows us to construct effective connectivity matrices that tell us how representations of being at a particular location at one time affect representations of being at a particular location at some other time.
We start by looking at the recurrent weights between subspaces that represent locations or expected future locations at adjacent moments in time.
The prediction from the STA is that this weight matrix will correspond to the adjacency matrix of the environment.
We compare three different models.
One is what we call the true STA, which are the actual parameters that we put into the handcrafted space-time attractor.

00:01:11
One is what we call the empirical STA, which is where we take the neural activity from the space-time attractor, the handcrafted space-time attractor, and we use this procedure to estimate effective connectivity, and we see that it works quite well.
And finally, we look at the task-optimized RNN, and we find that the learned connectivity closely resembles the adjacency matrix of the environment as predicted by the space-time attractor.
We then look at the input weights.
We've first looked at the inputs to the subspace that represents the current location of the agent, and the prediction from the STA is that the input should indeed be the current location of the agent.

00:01:46
We see that this is true both in the true STA, the empirical STA, and in the task-optimized RNN.
This is maybe not too surprising.
But we then look at the input to subspaces that represent the expected location at some point in the future.

The prediction from the STA is that this input should correspond to the reward function at that particular point in time.
And again, we find this is true in both the true STA, the empirical STA, and the task-optimized RNN.
So together, these analyses suggest that when you train neural networks to solve complex planning tasks, they learn to embed the structure of the world into their recurrent connectivity, which allows them to solve the problem using planning as inference, as in the space-time attractor.

00:02:25
We can also look at the recurrent connectivity between subspaces that represent locations at more distant points in time.
So in this case, we look at the recurrent weights from a direction of sp-state space that predicts being at a particular location in space at a particular moment in time, and we look at the effective weight matrix between this point in space-time and other points in space at different moments in time.
We see that the strongest component of the connectivity matrix is the adjacency matrix between these subspaces separated by one action, which is indicated by these green circles.

00:02:55
But there's also some weak connectivity to subspaces that represent more distant points in time, and we see that the structure of this connectivity again matches the structure of the world in that there are stronger connections to locations that can be reached in a particular number of actions.
This is slightly different from the handcrafted space-time attractor, but it's consistent with a neural network that's explicitly trained to solve planning by inferring future trajectories.
In pound E, we simply quantify this by doing a comparison of the effective connectivity between subspaces separated by different numbers of actions and matrices that indicate which locations are reachable in that particular number of actions.

00:03:36
Finally, we look at how this connectivity affects the dynamics of the network.
To do so, we consider a particular example environment which has two desirable trajectories, one of which is slightly better than the other.
We see that the trained RNN initially converges to a representation of the better path, which is what we would expect from the previous analyses.

But we then go and run a perturbation analysis.
In particular, we add an artificial input to a single point in space-time, which is a point in this alternative path.
We see that when this input or when this perturbation is weak, it doesn't affect the representation of the RNN at all, which suggests that this is a stable representation or a fixed point of the dynamics.

00:04:17
Conversely, when we pro-provide a very strong perturbation, we see that the entire representation shifts to a representation of the alternative path, even in subspaces not directly perturbed.
We can quantify this by asking how much does the representation change as a function of the stimulation strength?
And we see that weak perturbations are quenched, and then there's a near binary switch to a complete change in the representation towards this alternative path.
We can also look at how these representations change over time in the presence of perturbations of different strengths.

00:04:51
In this case, we see that initial activity is stable because it's at a fixed point of the recurrent dynamics.
When we add weak perturbation, the representation just stays the same.
But when we add strong enough perturbation, we see that this is changed to a representation of the alternative path in the task-optimized RNN.

One thing we also see is that after the end of the perturbation, the representation actually reverts back to the original fixed point.
What this tells us is that there's only one fixed point in the unperturbed dynamics, which is a representation of the better path.
But once you add a strong enough perturbation, you shift the attractor landscape such that the new fixed point is a representation of this alternative path.
