# Supplementary material for: A mechanistic theory of planning in prefrontal cortex
Source: eLife. 2026 Oct 21;15:RP109757. doi: 10.7554/eLife.109757 (PMC13686619; doi:10.7554/eLife.109757)
Supplement: Supplementary file 8 [file elife-109757-fig7.docx]

00:00:00
In this video, I explain figure seven, where we show how space-time attractors can generalize across environments with different structures.
Imagine you are this mouse trying to get to the cheese.
Depending on whether you are in a maze like this one or a maze like this one, you have to take very different sequences of actions to reach your goal.
This might seem incompatible with the idea that a space-time attractor builds the structure of the environment directly into the synaptic connections.
To investigate how networks can generalize across such environments, we trained a new set of RNNs that received a different configuration of walls on every trial, which was provided in their inputs.

00:00:35
We found that the network trained in a single environment failed to generalize to such new environments, but the network that was trained in changing environments performed almost as well in the single environment as the network that was specialized for that particular environment.
This raised the question of how these networks learn to generalize across environments.
So we went ahead and looked at whether they had explicit representations of the future and how these representations were connected.
Remarkably, we found that when we looked in a particular environment, and we looked at the connectivity, the effective connectivity between subspaces that represented adjacent times in the future, this effective connectivity matrix looked almost exactly like the adjacency matrix of that specific environment.

00:01:15
When we then took the same network, but we deployed it in a different environment, and we went and found the subspaces that represented expected locations at different times in the future, the connectivity between these new subspaces looked like the adjacency matrix of the new environment.
And we quantified this and showed that the effective connectivity between pairs of subspaces looks like the adjacency matrix or the structure of the environment that we used to identify these subspaces.
To understand how this is possible, we took inspiration from the fact that the subspaces change between environments.

00:01:47
So if we go and find the different subspaces or patterns of neural activity that predict future locations, either multiple times in the same environment or across different environments, we find that these subspaces or patterns of neural, neural activity are much more similar across different instances of the same environment.
So what's going on is that if you're in environment one, here represented in orange, then you might use one particular subspace to represent your next location and a different subspace to represent your location two actions, and these are connected according to a particular set of recurrent weights.
Now, if you move to a different environment, here illustrated in blue, you can use a different pattern of neural activity to represent where you'll be in one action and where you'll be in two actions.

00:02:30
And now, even though the, the true weight matrix of the RNN is the same, the effective weight matrix between these subspaces is going to be different because they align with different components of the complete connectivity matrix.
We can also understand how this works mechanistically.
And to do so, we have to generalize the idea of the space-time attractor from before slightly.
What we suggest is happening is that the network no longer represents just the location it will be at in a particular number of actions.
Instead, we think the RNN learns to represent what transition it will take in a particular number of actions, and that's what we illustrate in panel F.

00:03:07
In this case, you have individual neurons, here illustrated in the small circles, which represent a plan to move to the right from the central location in one action's time.
These neurons are still connected according to the structure of the environment.
So neurons that predict going up from the lower location in one action time will connect to other neurons that represent taking any action from the center location in two actions time, and so on and so forth.
This is really useful because it means that inputs can gate the effective connectivity of the circuit.

00:03:39
If it turns out there's a wall between these two locations here, you can simply inhibit all neurons that represent transitions across this edge of the graph, and it will be as if this connection never existed in the recurrent neural network.
And this allows you to generalize to any environment which can be constructed as some combination of the neurons that make up this general scaffold of Euclidean space.
So this model makes a couple of hypotheses that we went and tested in the task-optimized recurrent neural network.
One is that you should have these explicit representations of future transitions, which we show in a supplementary figure, but it also makes very precise predictions about the connectivity of the circuit.

00:04:19
First, it predicts that a representation of taking a particular transition in a number of actions should be excitatory connected to neurons that represent taking compatible transitions at the next moment in time.
But it should not be connected to neurons that represent taking incompatible transitions at the next moment in time.
And this is what we show in, in panel G.
And finally, we can make predictions about the inputs.
Because these networks receive an input that tells it where the walls will be in a particular environment, we know this input should inhibit all future representations of transitions through that particular wall, and this is what we show in panel H.
