# Supplementary material for: A mechanistic theory of planning in prefrontal cortex
Source: eLife. 2026 Oct 21;15:RP109757. doi: 10.7554/eLife.109757 (PMC13686619; doi:10.7554/eLife.109757)
Supplement: Peer review history

# Editor report: eLife Assessment

**DOI:** 10.7554/eLife.109757.3.sa0

**Authors:** Srdjan Ostojic

**Affiliations:** École Normale Supérieure - PSL France

**Keywords:** Compelling, Fundamental

This **fundamental** study advances our understanding of the neural substrate of planning trajectories towards a goal by using recurrent neural networks. The article provides **compelling** evidence for the proposed mechanism by linking a computational model to previous findings in neural data, designing a hand-crafted implementation of the mechanism, and showing that trained recurrent networks converge to computational principles consistent with the proposed mechanism. The work will be of broad interest to theoretical and systems neuroscientists and to cognitive scientists.

# Referee report: Reviewer #1 (Public review)

**DOI:** 10.7554/eLife.109757.3.sa1

Summary:

This work builds a theory to implement planning trajectories towards a goal in a known environment, inspired by analyses of prefrontal neural recordings. Unlike standard neural architectures for this task, such as value-based learning and successor representations, their proposed theory is able to adapt to novel goal locations within-trial. The key to the theory is that future times and locations are represented by disjoint groups of neurons. The recurrent connectivity between groups of neurons selective to specific future time and locations reflects the learned knowledge of the task. Finally, the authors show that standard networks trained on the task approximate their proposed theory.

Strengths:

The structure of the work is clear, and the article is very well written, which is particularly noticeable given the consequential amount of results presented. The authors are able to link their theory to experimental findings in neural recordings. The reverse-engineering of trained recurrent neural networks is very thorough, by analyzing both dynamics and connectivity. The assumptions and predictions of their model are clearly stated.

Weaknesses:

I believe the article shows no major weaknesses. There are important points that are beyond the scope of this study, that may limit its impact. For instance, while very little previous literature has linked planning to RNNs, their proposed theory of "space-time attractors" in RNNs is about input-driven stable attractors. The authors clarify this in the text, highlighting differences between classic attractor networks and the mechanism studied here. Related to this, an assumption of this mechanistic theory is that rewards are flexibly rerouted to the corresponding neural populations after every action, which seems difficult to implement biologically. Both aspects are discussed in the current manuscript.

# Referee report: Reviewer #2 (Public review)

**DOI:** 10.7554/eLife.109757.3.sa2

This well-written manuscript proposes to use attractors in space and time (STA) as a mechanistic explanation for planning in the prefrontal cortex. The main conceptual hypothesis is that planning is implemented as attractor dynamics in a representation that encodes states at each time step jointly. Depending on inputs the network relaxes to a trajectory that already contains future states that will be visited at each time step, rather than computing a scalar value at each point in time and space like other classical approaches from RL. The authors compare this approach to implementations such as TD learning and successor representation, and further show that trained recurrent neural networks on specific tasks involving planning develop structured subspaces resembling the ones postulated in STA.

The idea of treating attracting trajectories unfolding in time as the computational substrate for planning is very interesting and potentially important. The explicit construction of a state x time representational space and its implementation via recurrent dynamics are appealing and convincing in the idealized tasks considered. I found the ms to be refreshingly explicit regarding several of the assumptions and limitations of the models, for example the fact that certain advantages can be viewed as properties of the state space itself and not necessarily of a fundamentally new planning mechanism.

I thank the authors for their reply and their thorough rebuttal. It answered most of my previous questions and greatly enhanced the understanding of the paper.

I have just two remaining concerns:

(1) The ms shows attractor dynamics in the trained RNN during planning, but it is less clear how these relate to the execution phase. It would be helpful to clarify whether the network state during execution is expected to effectively be close to a FP or at a FP for each input, or whether the RNN implements transient dynamics shaped by the underlying attractor landscape.

(2) Regarding the previously raised point of calling their result a "Mechanistic theory of planning", I did not mean to suggest that a theory cannot be mechanistic, or that "mechanistic theory" is not a valid term, especially in the context of this paper (although I believe this topic would deserve an entire separate discussion in the neuroscience field).

My point was about whether STA should primarily be interpreted as a mechanistic theory of planning, or as a candidate neural mechanism for implementing the planning as inference theory. I am aware that mechanistic theory and mechanistic models are often used interchangeably in neuroscience, and I certainly do not claim that my interpretation is the only valid one. My opinion is that the manuscript presents a convincing and interesting candidate neural mechanism for planning, which can be strongly related to planning as inference. The reason why I am not fully convinced about the framing as a mechanistic theory of planning is mainly that the adjacency-based connectivity isn't emerging or derived, but is instead introduced based on practical and empirical considerations. It's not a major issue, but I would personally frame it as a mechanistic account or model of planning (and/or planning-as-inference), rather than a theory, mechanistic or not.

# Author comment: Author response

**DOI:** 10.7554/eLife.109757.3.sa3

**Authors:** Kristopher T Jensen, Peter Doohan, Mathias Sablé-Meyer, Sandra Reinert, Alon Boaz Baram, Maneesh Sahani, Thomas Akam, Timothy E Behrens

**Affiliations:** University College London London United Kingdom; University of Oxford Oxford United Kingdom; University College London London United Kingdom; University College London London United Kingdom; University of Oxford Oxford United Kingdom; University College London London United Kingdom; University of Oxford Oxford United Kingdom; University of Oxford Oxford United Kingdom

The following is the authors’ response to the current reviews.

We appreciate the additional clarifications suggested by the reviewers, and we will include these in the final Version of Record.

The following is the authors’ response to the original reviews.

**Reviewing Editor Comments:**

The reviewers are very enthusiastic about this study, but have pointed out a central issue: are "space-time attractors" really attractors?

The reviewers would be willing to increase the assessment of significance if the comments are properly addressed, and in particular, the issue about space-time attractors.

We thank the editors and reviewers for the feedback on our manuscript and have revised the paper to address their questions and concerns. This document includes (i) an overview of the major changes to the paper, and (ii) point-by-point responses to the reviewers. We have also attached a version of the revised paper that highlights substantial changes to the text.

Briefly, the reviewers asked for improved intuition about the STA representation and dynamics, and its relationship to attractor networks. To address their questions, we have restructured the paper. It now starts by introducing the STA, which has a representation and connectivity that are both handcrafted. The revised manuscript characterises the resulting dynamics and fixed points in more detail, both empirically and analytically. We then introduce a new model that directly optimises the fixed points of a neural network to represent an explicit plan of the future. The optimal weights for inferring such representations resemble the STA connectivity empirically. Finally, we analyse our unconstrained recurrent neural network, which learns both optimal representations and connectivity. As also shown in the original paper, this network learns to implement an algorithm that closely resembles an STA. Together, these results show that attractor networks can infer PFC-like representations of the future, and this is an efficient solution to dynamic planning problems known to depend on PFC.

RE1: Expanded theory of STA dynamics

We have now formalised how the STA relates to a formulation of planning as an inference process over future trajectories, which has been previously proposed in cognitive science and reinforcement learning. We show in the revised paper that the STA dynamics resemble an algorithm for approximate inference in the corresponding probabilistic graphical model. This allows us to characterise the fixed points of the algorithm analytically and relate them directly to a well-established cognitive theory of planning. These analyses help bridge the gap between neural implementation and cognitive computation. They shine new light on previous results in the paper while also providing more intuition for the STA dynamics.

We have also included a new model that directly optimises the fixed points of an attractor network to resemble a posterior distribution over future locations from planning-as-inference. This analysis complements the handcrafted STA, where we impose both the representation and connectivity, and the RNN, where both the representation and connectivity are learned. The new model imposes (i) an explicit spacetime representation, and (ii) the multiplicative structure of a message passing algorithm. We then train the weights associated with the forward and backward messages by gradient descent on the KL divergence between (i) the true posterior marginals and (ii) the approximate distribution over future locations implied by the network representation at the fixed point. Supplementary Figure S2 of the revised manuscript shows that the optimal weights reflect the transition structure of the environment, similar to the handcrafted STA model and the task-optimised RNN. This makes the connection between attractor dynamics and planning-as-inference more explicit by showing that the fixed points of an attractor network can be optimised directly for planning.

RE2: Improved characterisation of fixed points

We have clarified how and why the STA is an attractor network. Attractor networks are defined by the existence of stable fixed points. In ring and grid attractors, there is a continuum of such fixed points in the absence of structured inputs (but often with tonic excitation). In contrast, the STA has a discrete set of input-dependent fixed points. We show explicitly in the revised manuscript how these fixed points depend on the reward inputs to the network, and also how they relate to planning-as-inference.

We are not claiming that the STA is exactly equivalent to continuous ring and grid attractors. Instead, we want to convey the intuition that the connectivity of the STA constrains the possible fixed points to be plausible trajectories through space and time. The reward inputs determine which of these possibilities is an actual fixed point in a given planning problem. This is not unlike ring attractors in the presence of strong visual inputs. The connectivity enforces a single bump of activity, and the visual input ‘yokes’ the bump to an appropriate orientation. These similarities and differences are highlighted in the revised paper.

Finally, we have added a new Figure 3 to the main text that characterises the STA fixed points empirically. This figure:

(a) Shows the evolution of the STA dynamics and convergence to different fixed points in different environments (panels A-B).

(b) Shows that the network can converge to different fixed points on different trials in the same environment. This happens when there are multiple equally good paths to a goal (panels B-D).

(c) Shows that other fixed points also exist that correspond to longer trajectories, but the dynamics of the network bias it towards representations of shorter paths. The STA reliably converges to fixed points representing longer trajectories if it is initialised within their basin of attraction (panel F).

Updated main text:

“Unlike ring and grid attractors, the fixed points of the spacetime attractor depend on tonic inputs. However, the connectivity constrains the fixed points to represent continuous trajectories for any combination of inputs. In this section, we show this empirically. Later, we will see that such connectivity is optimal for planning-as-inference.

To compute a plan, it is necessary to know which states will be rewarding in the future. This reward information is provided as an input to the STA and enables fast adaptation without rewiring the synaptic connections. It alters the fixed points of the recurrent dynamics to only include trajectories that are also associated with high cumulative reward (Figure 3; Methods).”

RE3: Ground truth rewards as an input to the network

Both reviewers asked about the external input to the STA that specifies the reward available at different states in the future. In reinforcement learning and cognitive science, ‘planning’ is usually defined as the problem of computing a trajectory that maximises cumulative future reward, given an initial state and a reward function (e.g. Mattar & Lengyel, 2022). This is similar to many real-life situations, where we have a known but distant goal (win a game of chess, finish our paper before a deadline, …). When such a reward function is known, it remains challenging to determine the sequence of actions to get there. This has been the topic of much previous work in neuroscience, including (i) the successor representation, which combines a trial-specific reward function with stable transition statistics; and (ii) different types of sequential search, which use a known reward function to evaluate different possible future trajectories.

To highlight the importance of planning, even when the reward function is known, Figure 4 of the revised manuscript shows that the STA performs better than a greedy baseline that acts according to the immediate reward input instead of planning to maximise cumulative reward. Planning is therefore distinct from learning or inferring a reward function, which is itself a major open question in cognitive science. While undoubtedly interesting, a solution to this problem is beyond the scope of our paper. That is why we decided to simply provide ground truth rewards as an input to the STA. We have clarified the distinction between planning and ‘reward learning’ in the revised paper, and the supplementary material now includes a discussion of where the reward input to the STA could come from.

**Author response image 1.** All performance quantifications in Figure 4 now include an additional ‘greedy’ baseline (grey bars). This is an agent that acts according to the immediate future reward. The performance improvement of the STA over this baseline highlights the importance of planning to maximise cumulative reward.

Updated main text:

“There are several possible sources of reward input to a spacetime attractor (Supplementary Note). We focus on planning under a known reward function and therefore assume access to ground-truth rewards.”

**Reviewer #1 (Public review):**

Summary:

This work builds a theory to implement planning trajectories towards a goal in a known environment, inspired by analyses of prefrontal neural recordings. Unlike standard neural architectures for this task, such as value-based learning and successor representations, their proposed theory is able to adapt to novel goal locations within a trial. The key to the theory is that future times are represented by orthogonal groups of neurons. The recurrent connectivity between groups of neurons selective to specific future times and locations reflects the learned knowledge of the task. Finally, the authors show that standard networks trained on the task approximate their proposed theory.

Strengths

The structure of the work is clear, and the presentation of the results is very well written, which is particularly noticeable given the consequential amount of results presented. The authors are able to link their theory with experimental findings in neural recordings. The reverse-engineering of trained recurrent neural networks is very thorough, by analyzing both dynamics and connectivity. The assumptions and predictions of their model are clearly stated.

We appreciate the encouraging comments and hope our revised manuscript addresses the reviewer’s questions.

Weaknesses

(1.1) It is unclear whether their proposed theory, "space-time attractors", actually is an attractor network. The authors used recurrent neural networks with very few timesteps, and long single neuron time constants with respect to the task time scales. Attractor networks, as the ones the authors cite, refer to networks that generate nontrivial patterns of activity through recurrent interactions, after long periods of time.

See RE1 & RE2 for a comprehensive response to this question. Briefly, we show in the revised manuscript how the fixed points of the STA dynamics relate to planning-as-inference, and we clarify the similarities and differences between the STA and other attractor networks in the main text. We show in the new Figure 3 that (i) representations of future paths are stable over long periods of time, and (ii) multiple fixed points can exist when there are multiple paths to the goal. It is also worth noting that the RNN representation in Figure 6H remains stable for 75 time constants and recovers from perturbations. This is substantially longer than during training, where ‘planning’ lasted up to 14 network time constants, and it suggests that the network representation is a stable fixed point.

(1.2) The authors gloss over how the reward inputs are calculated. Computing these reward inputs should be part of the planning process, and the authors are implicitly leaving this problem aside. How does the reward input, which includes future time and location, depend on the actions that have not yet been taken by the agent? It feels like most of the planning computation is already provided by these reward inputs at the beginning of the trial. It could be that the network is only learning to process the planned sequence of actions present in the inputs.

See RE3 for a comprehensive response to this question. Briefly, ‘planning’ is often defined as the problem of computing a trajectory that maximises future reward, given a reward function, initial state, and transition function. The reward function provided to the agent indicates which future states it would be desirable to reach, but not how to reach them. To make this point clearer, we show in Author response image 1 that the representations computed by the STA generate better behaviour than an agent acting greedily according to the reward function specified by the inputs. This highlights the importance of considering distant goals when choosing immediate actions.

**Reviewer #1 (Recommendations for the authors):**

The text is very nicely written, and I appreciated the way in which methods are presented, with a clear structure and a logical chaining of the different sections. My comments and suggestions refer mostly to the methods and the RNN implementation. Please find below a list of issues.

Relatively major:

(1.3) All the equations of the dynamics should be written in discrete time and not in continuous time. There is no notion of "iteration" in continuous time, so it is currently very hard to understand how the RNN works, and what the different epochs are ("the RNN performed 10 network iterations..."?).

We have rewritten all equations in discrete time and clarified the notion of ‘iterations’.

(1.4) This is pointed out in the public review, but the authors insist on making an analogy between the spacetime attractor implementation of planning and attractor networks. It seems to me that these two types of models are very different. What defines attractor networks (such as grid- or ring-attractor networks) is that recurrent connections internally generate stable states of activity for long periods of time, in the absence of inputs. Nothing like that is shown here. Robust input-driven trajectories are neither necessary nor sufficient for showing that an RNN is an attractor network. The fact that, given the inputs, networks are run for very short periods of time in this work seems to indicate that this is a very different type of network compared to the attractor networks mentioned previously.

See RE1 & RE2 for a comprehensive response to this question. Briefly, it is correct that the fixed points of the STA depend on the inputs, which is different from canonical ring and grid attractors.

We show that the fixed points of the recurrent STA dynamics are reward-maximising paths when conditioned on those inputs. Briefly, we now (i) analytically characterise the input-dependence of the fixed points and show how they relate to planning-as-inference; and (ii) show empirically that STA representations remain stable for long periods of time (Figure 3). The revised paper also clarifies the similarities and differences to previous attractor models.

Minor

(1.5) It would be nice to show more clearly what the inputs are in a given trial, and how they change over time in the RNN (specifying the planning and execution phases). A supplementary figure may help.

How is the information about walls provided to the network exactly? More generally, it would be nice to clearly indicate in the Methods what all the inputs "x" to the RNN are, and how they change over time (during planning, during execution, and how they change as the environment steps are updated).

We have made a new Supplementary Figure S3 that illustrates the inputs to and outputs from the different models. Briefly, the information about the walls is provided to the RNN as a binary vector *x_w_* ∈ ℝ ^2*𝑁*^. The elements of this vector indicate for each of the N states whether there is a wall (i) to the right of, and (ii) above it. In each trial, a subset of these is present, and a subset is absent. All ‘present’ walls are assigned a value of +1 in *x_w_* , and all absent walls are assigned a value of 0 in *x_w_* . We have also clarified this in the revised Methods.

(1.6) It would help to clarify, at least in the methods, the shape of all the matrices and vectors that are trained.

We have clarified the shapes of all matrices and vectors in the Methods.

(1.7) N in the methods is not defined (I think N = 16, the total number of locations on the grid).

N is indeed the total number of locations in the state space. This is 16 for almost all analyses in the paper, which involve planning on a 4x4 grid. The updated manuscript includes a few analyses in larger environments, where N is larger. We have clarified this in the Methods.

**Reviewer #2 (Public review):**

This well-written manuscript proposes to use attractors in space and time (STA) as a mechanistic explanation for planning in the prefrontal cortex. The main conceptual hypothesis is that planning is implemented as attractor dynamics in a representation that encodes states at each time step jointly. Depending on inputs, the network relaxes to a trajectory that already contains future states that will be visited at each time step, rather than computing a scalar value at each point in time and space like other classical approaches from RL. The authors compare this approach to implementations such as TD learning and successor representation, and further show that trained recurrent neural networks on specific tasks involving planning develop structured subspaces resembling the ones postulated in STA.

The idea of treating attracting trajectories unfolding in time as the computational substrate for planning is very interesting and potentially important. The explicit construction of a state x time representational space and its implementation via recurrent dynamics are appealing and convincing in the idealized tasks considered. I found the manuscript to be refreshingly explicit regarding several of the assumptions and limitations of the models, for example, the fact that certain advantages can be viewed as properties of the state space itself and not necessarily of a fundamentally new planning mechanism.

Overall, the manuscript presents a cool attractor model that extends in time and explores its performance in a subset of illustrative tasks involving planning. My doubts concern mostly the interpretation and scope of the claims made in the manuscript. Here are a few comments where I detail my questions/concerns:

We appreciate the enthusiasm about the manuscript and its potential importance. We address the remaining questions and concerns below.

(2.1) The authors nicely discuss that much of the difference between STA and classical TD or SR agents is "in some sense a property of the state space rather than the decision making algorithm," and that TD and SR could in principle be implemented in a comparable space x time representation. This is fair, but it also suggests that the central contribution of the manuscript lies primarily in the representational factorization (state x time tiling) and its dynamical implementation via attractors, rather than in a fundamentally new planning algorithm or theory, mechanistic or not. I think theory should be distinguished from mechanism, and it would therefore help the reader to describe the conceptual advancement more as a novel mechanism or implementation than a novel (mechanistic) theory for decision/planning.

We respectfully disagree that ‘theory’ has to be distinguished from ‘mechanism’. We do agree that ‘computation’ and ‘mechanism’ can often be distinguished. However, we think theories can live at either of these (and other) levels of explanation. What we propose is indeed a potential mechanism for planning that combines recently characterised prefrontal spacetime representations with attractor dynamics to infer desirable ‘plans’. As we show in the revised manuscript, this mechanism resembles the computation of ‘planning-as-inference’, which has previously been proposed in cognitive science (e.g. Botvinick & Toussaint, 2012). Our theory is therefore not about the computation – it is about the mechanism. The title “A mechanistic theory of planning…” is meant to clarify what level of description our paper addresses.

As an example of the importance of mechanistic theories, the computation of angular velocity integration can be implemented in many different ways. Seminal work by Skaggs et al. (1994) and others in the 1990s showed how it can be implemented in neural networks, inspired by experimental data. These theories paved the way for detailed experimental characterisations of the fruit fly head direction circuit more than two decades later (Turner-Evans et al., 2017; Kim et al, 2017; and others). Inspired by this and other success stories, we think an important role of theoretical neuroscience is to develop theories about neural mechanisms that can be tested in future experiments!

(2.2) Related to my previous point, I think it would be helpful to position STA more explicitly relative to computational/theoretical literature in which attractor networks encode temporally ordered patterns (so effectively including future times). For example, classical extensions of Hopfield networks with asymmetric connectivity implement retrieval of sequences and ordered transitions between patterns (Sompolinsky & Kanter, 1986). More recently, sequential attractors and limit-cycle dynamics have been constructed in structured recurrent networks by the Morrison group (Parmelee et al., 2021). These works do not implement an explicit discretized state x future-time tiling as in STA and do not specifically discuss the usage for planning. However, they do provide concrete precedents for attractor dynamics over temporally structured trajectories in terms of mechanism. It would be useful to discuss this literature and clarify a little what's new mechanistically in the view of the authors.

We agree that this is not the first use of attractor networks to represent or compute sequences. Instead, we show that a combination of spacetime representations with attractor dynamics is sufficient to compute plans in dynamic problems known to depend on prefrontal cortex. As the reviewer points out, the primary difference from most previous work lies in the fact that the entire sequence is encoded in a single fixed point of the STA dynamics. This differs from e.g. Sompolinsky & Kanter, where the population encodes one element at a time and generates sequences as limit cycles. The instantaneous encoding of an entire sequence in the STA is what enables planning through parallel message passing rather than sequential search. This is highlighted in the main text of the revised manuscript, which also includes a supplementary discussion of the similarities and differences between the STA and related work on sequences in attractor networks.

Updated main text:

“Entorhinal grid cells are also embed a world model in their connectivity (McNaughton et al., 2006), but they only encode a single location at a time (Vollan et al., 2026). Such networks can generate sequences, but the individual elements are represented one by one (Sompolinsky and Kanter, 1986; Kleinfeld, 1986; Widloski et al., 2025). The spacetime attractor suggests that circuit principles in prefrontal cortex resemble other cortical areas that use structural knowledge to infer features of the world. The major difference is that PFC instantaneously represents many points in time, which generalises known circuit principles to complex planning.”

(2.3) A central claim of the manuscript is that space-time trajectories are attractors of the STA dynamics. The manuscript does provide empirical evidence consistent with attractor-like behavior. However, it is not explicitly shown whether trajectory representations persist in the absence of sustained external inputs. So it's not clear to me whether the trajectories should be interpreted as intrinsic attractors of the recurrent system, which can be selected by delivering transient inputs, or whether they must be stabilized by a specific continuous external drive. It would be useful if the author could clarify/discuss this point.

We show in the revised paper that the fixed points of the STA dynamics take the form *r _δ_* = *e^R_δ_^* ◦ (*Ar_δ−1_*) ◦ (A^T^ *r_δ+ 1_*) (Methods). Here, *r* δ is the activity of neurons representing expected locations in δ actions; *R* δ is the reward function in δ actions; and *A* is the environment adjacency matrix. These fixed points depend on the reward inputs through the first term. In the absence of reward inputs, the fixed points are ‘diffusive’, while still respecting the transition structure of the environment. In the presence of reward inputs, they concentrate probability mass on trajectories with high expected reward. We have clarified these properties in the main text and introduced a new Figure 3 that characterises the fixed points of the STA in more detail. See also RE1 and RE2.

(2.4) As far as I understand it, reward information is provided as input to specific populations encoding future time steps, and that's essential for rapid adaptation without rewiring connectivity. How such future-time-specific reward inputs would be generated and routed to distinct neural populations isn't entirely clear to me. Since this seems to be an essential component of the model, I think it would be important to discuss more deeply the source and plausibility of these reward signals related to different timesteps.

See RE3 for a comprehensive response to this question. Briefly, ‘planning’ is often defined as the problem of computing a trajectory that maximises future reward, given a reward function, initial state, and transition function. The reward function provided to the agent indicates which future states it would be desirable to reach, but not how to reach them (see Author response image 1). We agree that the challenge of estimating future reward is an interesting question, but it is beyond the scope of this paper. We have clarified this distinction in the main text and added a supplementary discussion that speculates about where reward information could originate in biological circuits.

(2.5) The authors note that vanilla STA scales linearly with planning horizon, and discuss potentially hierarchical extensions for longer horizons. They acknowledge that learning abstractions remains an open challenge, yet the examples of planning in the manuscript are restricted to very short temporal horizons and limited branching complexity. It is not obvious to me in what cases the current implementation and interpretation of STA remains viable (for example, in terms of relaxation iterations) as the horizon and branching factor increase. Relatively simple planning can be managed by simpler, less costly models/algorithms, whereas complex planning is a lot harder to deal with, and it's something that a mechanistic "theory" should address. In the context of the claims of the paper in its present form, I think this is possibly the most important conceptual and practical limitation in the manuscript.

It is correct that planning gets increasingly challenging with planning depth. In the absence of noise, the STA scales to sequences of up to 12-13 actions – and even longer if the minimum path length is known a priori*.* Performance gets progressively worse when recurrent activity and parameter noise increase. The revised paper includes a new Supplementary Figure S1A-C that shows how the STA planning ability depends on planning depth for different levels of noise.

We do not consider planning depth to be a major limitation of the work, since humans are rarely thought to plan much more than 6 steps into the future at a single level of abstraction (e.g. van Opheusden et al., 2023). Instead, we believe that hierarchical planning is used to infer trajectories to distant goals (Eckstein & Collins, 2020). To illustrate this point, we have now implemented a proof-of-principle hierarchical STA in Supplementary Figure S1D-E. This simulation shows how an ‘abstract plan’ inferred by one STA can be treated as a goal to infer a more ‘detailed plan’ in a second STA. In principle, this enables the system to compute plans that are arbitrarily long, provided they can be broken down into chunks smaller than the limits imposed by the analyses in Supplementary Figure S1A-C.

Finally, RNNs learn an STA-like algorithm when trained on dynamic planning problems with a planning depth of 6. It is therefore not clear to us whether simpler and less costly algorithms can be easily implemented in the dynamics of recurrent networks.

(2.6) The RNN analyses show that trained networks develop structured subspaces aligned with future time indices and exhibit perturbation behavior consistent with attractor-like dynamics. The manuscript also explicitly notes differences between the trained RNN and the handcrafted STA (e.g., long-range couplings between subspaces and differences in behavior of lower-value trajectories under perturbation), which I much appreciated. My doubt is on the specificity of this result, as trained RNNs on fixed-horizon tasks can develop latent dimensions correlated with temporal progress within a trial or time-to-goal. I think it would help the reader to clarify whether the results demonstrate that STA-like computations emerge in RNNs trained on planning tasks, or that RNNs generally develop some kind of structured spacetime representations when tasks involve future timesteps and some degree of flexibility in the decisions.

An important point to note is that the subspaces we identify do not encode time-to-goal, since they are all active at the very beginning of the trial. We also show that RNNs trained on simpler static tasks do not learn the same algorithm (Supplementary Figure S7) and do not generalise to dynamic problems (Figure 5F). Finally, other algorithms are capable of solving the dynamic problems we study (e.g. the ‘value agent’ in Figure 5B-E). We therefore do not think it is trivial that RNNs learn an STA-like algorithm.

We do think that ‘structured spacetime representations’ generally emerge in RNNs trained on tasks that involve flexible behaviour in changing environments – in some sense that is the claim we are trying to make. It is known that spacetime representations are optimal for structured sequence memory tasks (e.g. Whittington et al., 2025; Dorrell et al., 2026), and we think this is for exactly the same reason. In sequence working memory, the reward function changes in time – for each action, the reward is only non-zero at the corresponding sequence element. However, the adjacency matrix is uniform for sequence memory – any sequence element can follow any other sequence element – so there is no need for planning. We are therefore not claiming that spacetime representations only emerge in the specific planning task we consider here. Instead, we expand the set of problems solvable by such representations to also include adaptive planning known to depend on prefrontal cortex. We have made this more explicit in the revised manuscript.

Updated main text:

“Together, our analyses show that RNNs trained on a dynamic planning task learn to approximate a spacetime attractor. This was also true across variations in model architecture (Methods; Figure S10; Figure S11). These results extend previous findings that explicit spacetime representations are optimal for sequence memory (Supplementary Note; Whittington et al., 2023; Dorrell et al., 2026; Wang et al., 2025). Additionally, RNNs with too few hidden units to learn a spacetime attractor failed to solve the task (Figure S12), suggesting that other solutions are not readily learned by gradient descent.”

A few more minor points, mainly concerning clarity:

(2.7) The main dynamical equation combines a log-domain recurrent term, a floor operation, and a log-sum-exp normalization step, followed by exponentiation. The intuition/logic behind this specific formulation could be clarified for the reader. For example it would be helpful to explain why the recurrent input appears inside a log, and also whether/how these operations relate to any multiplicative constraint.

The specific form of these equations comes from the intuition that the STA approximates planning as an inference process over future trajectories. We have clarified this in the revised manuscript, which explicitly shows how these equations relate to planning-as-inference as formulated previously (e.g. Botvinick & Toussaint, 2012; Levine, 2017).

(2.8) While the computational cost of successor representation in an expanded NT x NT representation is discussed, the corresponding scaling of STA in terms of number of units and connections (as a function, for example, of the planning horizon) isn't clear to me. Perhaps the authors could compare costs more explicitly.

The memory cost of a spacetime-SR would be (NT)^2 and the computational cost (NT)^3 (it is possible that both of these could be reduced by taking advantage of the structured nature of the spacetime successor matrix, but that is beyond the scope of this work). The memory cost of the STA is NT, and the computational cost is (NT)^2 (each iteration of the network dynamics requires the calculation of T matrix-vector products of size NxN, and the number of steps to convergence is approximately linear in T). We have included this comparison in the Supplementary Discussion of the revised paper.

(2.9) In the RNN analyses, structured subspaces aligned with future time indices are shown. I couldn't find a quantification of how much variance is captured by the subspaces, relative to other latent dimensions. Adding it would help get a feeling for the strength of the alignment.

We have added a new Supplementary Figure S6 to the revised manuscript, which quantifies the variance explained by the future-coding subspaces over the course of a trial. The variance explained by the K dimensions encoded by these subspaces is substantially higher than a random baseline, and it approaches the upper bound given by the top K PCs. Interestingly, the future-coding subspaces all explain a lot of variance early in the execution period. During later stages of execution, only the ‘immediate future’ subspaces explain substantial variance. This suggests that the RNN only maintains information in subspaces that represent times before the end of the trial.

References

Botvinick, Matthew, and Marc Toussaint. "Planning as inference." Trends in cognitive sciences 16.10 (2012): 485-488.

Dorrell, William, et al. "An Efficient Computing Theory of Prefrontal Structured Working Memory Representations." bioRxiv (2026): 2026-02.

Eckstein, Maria K., and Anne GE Collins. "Computational evidence for hierarchically structured reinforcement learning in humans." Proceedings of the National Academy of Sciences 117.47 (2020): 29381-29389.

Kim, Sung Soo, et al. "Ring attractor dynamics in the Drosophila central brain." Science 356.6340 (2017): 849-853.

Levine, Sergey. "Reinforcement learning and control as probabilistic inference: Tutorial and review." arXiv preprint arXiv:1805.00909 (2018).

Mattar, Marcelo G., and Máté Lengyel. "Planning in the brain." Neuron 110.6 (2022): 914-934.

Skaggs, William, et al. "A model of the neural basis of the rat's sense of direction." Advances in neural information processing systems 7 (1994).

Turner-Evans, Daniel, et al. "Angular velocity integration in a fly heading circuit." Elife 6 (2017): e23496.

Van Opheusden, Bas, et al. "Expertise increases planning depth in human gameplay." Nature 618.7967 (2023): 1000-1005.

Whittington, James CR, et al. "A tale of two algorithms: Structured slots explain prefrontal sequence memory and are unified with hippocampal cognitive maps." Neuron 113.2 (2025): 321-333.
